# Can Gram-Negative Bacteria Develop Resistance to Antimicrobial Blue Light Treatment?

**DOI:** 10.3390/ijms222111579

**Published:** 2021-10-27

**Authors:** Aleksandra Rapacka-Zdonczyk, Agata Wozniak, Beata Kruszewska, Krzysztof Waleron, Mariusz Grinholc

**Affiliations:** 1 Department of Pharmaceutical Microbiology, The Faculty of Pharmacy, Medical University of Gdansk, Hallera 107, 80-416 Gdansk, Poland; beata.kruszewska@phdstud.ug.edu.pl (B.K.); krzysztof.waleron@gumed.edu.pl (K.W.); 2 Laboratory of Photobiology and Molecular Diagnostics, Intercollegiate Faculty of Biotechnology, University of Gdansk and Medical University of Gdansk, Abrahama 58, 80-307 Gdansk, Poland; agata.wozniak@phdstud.ug.edu.pl (A.W.); mariusz.grinholc@biotech.ug.edu.pl (M.G.)

**Keywords:** antimicrobial blue light, resistance, tolerance, *Escherichia coli*, *Klebsiella pneumoniae*, *Pseudomonas aeruginosa*

## Abstract

Antimicrobial blue light (aBL) treatment is considered low risk for the development of bacterial resistance and tolerance due to its multitarget mode of action. The aim of the current study was to demonstrate whether tolerance development occurs in Gram-negative bacteria. We evaluated the potential of tolerance/resistance development in *Escherichia coli*, *Klebsiella pneumoniae*, and *Pseudomonas aeruginosa* and demonstrated that representative Gram-negative bacteria may develop tolerance to aBL. The observed adaption was a stable feature. Assays involving *E. coli* K-12 *tolC*-, *tolA-*, *umuD-*, and *recA*-deficient mutants revealed some possible mechanisms for aBL tolerance development.

## 1. Introduction

One of the most urgent concerns in global healthcare in the 21st century is increasing resistance to antibiotics. The overuse and misuse of antibiotics has resulted in the occurrence of extensive drug-resistant strains (XDR). According to the U.S. Centers for Disease Control and Prevention (CDC), 700,000 people worldwide, including nearly 60,000 newborns in India, die annually due to infections that are caused by multidrug-resistant pathogens. It is estimated that this number will increase to 10 million by 2050 [1]. For this reason, in 2017, the World Health Organization (WHO) released a priority list of antibiotic-resistant pathogens. The majority of the pathogens that are on the list are Gram-negative bacteria, which are considered more resistant than Gram-positive bacteria due to their elaborate cell wall structure. The outer membrane of Gram-negative bacteria is considered the main reason for their multidrug resistance. Therefore, new antimicrobial approaches are still in demand to control this global health crisis [2]. One such innovative non-antibiotic approach is inactivation by antimicrobial blue light (aBL), which has a wavelength spectrum ranging from 400 to 470 nm [3]. Recently, aBL inactivation effectiveness and mechanisms of action have been extensively studied [4,5,6,7,8,9,10]. aBL inactivation could be a promising therapeutic option for the treatment of hospital-acquired infections that are caused by *Enterococcus faecium*, *Staphylococcus aureus*, *K. pneumoniae*, *Acinetobacter baumannii*, *P. aeruginosa*, and *Enterobacter* species (ESKAPE pathogens). These microorganisms rapidly develop resistance and avoid the effects of antimicrobial drugs, which makes infections with these pathogens especially challenging to treat. aBL inactivation has been shown to effectively eradicate ESKAPE pathogens [11,12]. The high efficiency of aBL inactivation is thought to be based on a multitarget mode of action that is caused by the rapid reaction of singlet oxygen with a wide range of cellular macromolecules, such as nucleic acids, proteins, and lipids, causing bacterial cell damage and death [13,14]. However, the detailed mechanisms remain unclear [15]. The proposed hypothesis is that aBL involves photoactivable endogenous metal-free porphyrins that naturally accumulate in bacterial cells [16,17].

Two phenomena are responsible for the failure of antimicrobial treatment: resistance and tolerance. Resistance is an acquired and inherited decline in the effectiveness of a given treatment, where higher concentrations of antimicrobial agents are needed to obtain the same bactericidal efficacy, while tolerance, which is a more general term, requires a prolonged treatment duration for a cure [18,19].

Due to the multitarget mode of action, antimicrobial photodynamic inactivation (including aBL inactivation) treatment is considered low-risk for the development of resistance. In contrast to antibiotics, full resistance to phototreatment (antimicrobial photodynamic [aPDI] and aBL inactivation) has not been reported. However, some recent reports have indicated that the development of a stable tolerance to aBL could be initiated by sublethal photoinactivation in some Gram-positive species. Our recently published studies [20,21] revealed that sublethal phototreatment lead to a tolerance in Gram-positive species (i.e., *S. aureus*, *E. faecium*, and *Streptococcus agalactiae*), due to an increased mutation rate directly resulting from the reactive oxygen species (ROS)-induced DNA damage and increased the activity of the stress-responsive error-prone DNA polymerase V [20]. Snell et al. confirmed a stable tolerance development against methylene blue-mediated antimicrobial photodynamic inactivation (575–700 nm) [22]. Nonetheless, previous research on Gram-negative species has not indicated adaptation to photoinactivation. For example, evaluation of the potential for resistance development in clinical isolates of *E. coli*, *P. aeruginosa*, and *A. baumannii* did not report any statistically significant changes in susceptibility to aBL [16,23,24]. To the best of our knowledge, the development of tolerance to aBL has not yet been described in Gram-negative species, and this is the first report of this phenomenon.

*Enterobacteriaceae* family pathogens (i.e., *E. coli* and *K. pneumoniae*), are the primary etiological factors for urinary tract infections (UTIs), hospital-associated pneumonia, and sepsis. Various mechanisms induce resistance to antimicrobials that lead to multidrug resistance (MDR), predominantly the production of extended-spectrum beta-lactamases (ESBLs). *P. aeruginosa* is an aerobic bacterium that, in addition to being part of the physiological flora of the intestinal tract, is also a health-threatening pathogen; it is the fourth most frequently isolated hospital-acquired microorganism. *P. aeruginosa* causes, among other things, intensive care unit (ICU)-acquired infections in seriously ill or immuno-compromised patients. Antibiotic resistance is supported by miscellaneous innate and acquired mechanisms [25].

This study aimed to assess the crucial risk of tolerance/resistance development that is associated with this new antimicrobial approach. Therefore, the study’s aim was to determine whether multiple sublethal exposures to aBL lead to tolerance or resistance development in the Gram-negative representative bacteria, *E. coli*, *K. pneumoniae*, and *P. aeruginosa*.

## 2. Results

### 2.1. Determination of the aBL MDK_99_ Conditions

We proposed a protocol to examine potential bacterial tolerance and resistance development that was based on the application of sublethal doses or irradiation times no longer than the MDK_99_. The aBL treatment conditions that caused an approximately 2 log_10_ decrease in the viable count for each strain are presented in Table 1.

### 2.2. Tolerance Development Was Induced in All Tested Gram-Negative Species

A significant tolerance development was observed in *E. coli*, *K. pneumoniae*, and *P. aeruginosa*.

#### 2.2.1. *E. coli*

**Figure 1:**
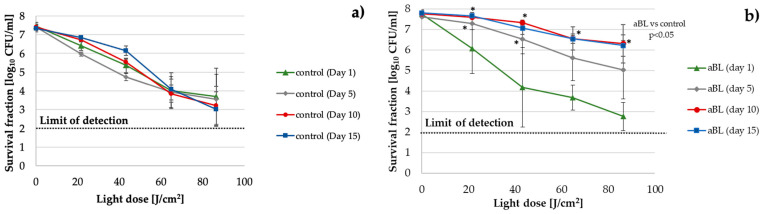
*E. coli* K-12 aBL tolerance development upon exposure to sublethal aBL treatment. Overnight *E. coli* K-12 cultures (37 °C in LB for 16–20 h) were diluted to an OD of 0.5 McF, and 100-µL samples were irradiated with 415 nm light at a dose of 32.4 J/cm^2^ (MDK_99_). Following exposure, 10-μL aliquots of the treated samples were collected to determine the survival rate. A total of 50-μL of the sample was transferred into fresh LB medium (5 mL) to regrow overnight. The next day, after 16–20 h of incubation, the treatment was repeated under the same conditions. The cycle of exposure—regrowth—exposure was repeated 15 times. The potential reductions in the susceptibility of *E. coli* K-12 to aBL were examined after the 5th, 10th, and 15th consecutive cycles at higher doses of light (up to 86.4 J/cm^2^) (**a**). The efficacy of aBL was also tested in samples from the untreated controls (these were passaged daily without the selective pressure of aBL) (**b**). The detection limit was 100 CFU/mL. The values are the means of three separate experiments. The error bars represent the standard deviations (SDs). * All of the significant differences were estimated in comparison to the control from each day.

An approximately 3 log_10_ unit decrease in aBL antimicrobial effectiveness was observed starting in the fifth cycle of the consecutive treatments as compared to the control, which was passaged daily without the selective pressure of aBL (Figure 1). An increasing decline in efficiency was observed through the 10th cycle, and by the 15th cycle, it remained at a constant level.

#### 2.2.2. *K. pneumoniae*

**Figure 2:**
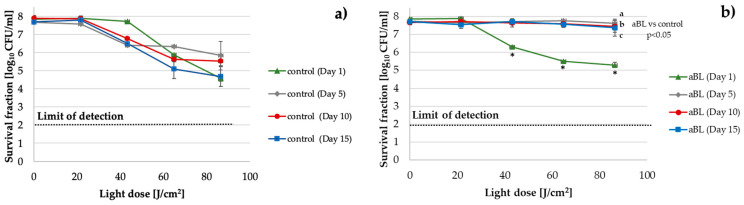
*K. pneumoniae* (ATCC700603) aBL tolerance development upon exposure to sublethal aBL treatment. Overnight *K. pneumoniae* cultures (37 °C in LB for 16–20 h) were diluted to an OD of 0.5 McF, and 100-µL samples were irradiated with 415 nm light at a dose of 61.2 J/cm^2^ (MDK_99_). Following exposure, 10-μL aliquots of the treated samples were collected to determine the survival rate. A total of 50-μL of the sample was transferred into fresh LB medium (5 mL) to regrow overnight. The next day, after 16–20 h of incubation, the treatment was repeated under the same conditions. The cycle of exposure—regrowth—exposure was repeated 15 times. The potential reductions in the susceptibility of *K. pneumoniae* to aBL were examined after the 5th, 10th, and 15th consecutive cycles at higher doses of light (up to 86.4 J/cm^2^) (**a**). The efficacy of aBL was also tested in samples from untreated controls (passaged daily without the selective pressure of aBL) (**b**). The detection limit was 100 CFU/mL. The values are the means of three separate experiments. The error bars represent the standard deviations (SDs). ^a^—aBL (Day 5) vs. control (Day 5), ^b^—aBL (Day 10) vs. control (Day 10), ^c^—aBL (Day 15) vs. control (Day 15)—significant results indicated for light doses ranging from 43.2 J/cm^2^ to 86.4 J/cm^2^. * All of the significant differences were estimated in comparison to the control from each day.

An approximately 2 log_10_ unit decrease in aBL antimicrobial effectiveness was observed starting in the fifth cycle of the consecutive treatments when compared to the control, which was passaged daily without the selective pressure of aBL (Figure 2) and remained relatively constant during consecutive cycles.

#### 2.2.3. *P. aeruginosa*

**Figure 3:**
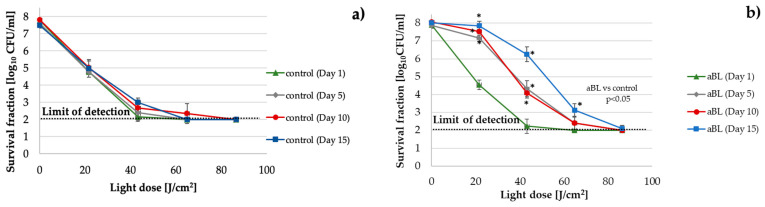
*P. aeruginosa* P14 aBL tolerance development upon exposure to sublethal aBL treatment. Overnight *P. aeruginosa* cultures (37 °C in LB for 16–20 h) were diluted to an OD of 0.5 McF, and 100-µL samples were irradiated with 415 nm light at a dose of 6.4 J/cm^2^ (MDK_99_). Following exposure, 10-μL aliquots of the treated samples were collected to determine the survival rate. A total of 50-μL of the sample was transferred into fresh LB medium (5 mL) to regrow overnight. The next day, after 16–20 h of incubation, the treatment was repeated under the same conditions. The cycle of exposure—regrowth—exposure was repeated 15 times. The potential reductions in the susceptibility of *P. aeruginosa* to aBL were examined after the 5th, 10th, and 15th consecutive cycles at higher doses of light (up to 86.4 J/cm^2^) (**a**). The efficacy of aBL was also tested in samples from untreated controls (passaged daily without the selective pressure of aBL) (**b**). The detection limit was 100 CFU/mL. The values are the means of three separate experiments. The error bars represent the standard deviations (SDs). * All of the significant differences were estimated in comparison to the control from each day.

An approximately 2 log_10_ unit decline in aBL antimicrobial efficacy was observed starting in the fifth cycle of the consecutive treatments compared to the control, which was passaged daily without the selective pressure of aBL (Figure 3). The decline in the efficiency was comparable in cycles 5 and 10, and in cycle 15, the efficiency dropped significantly (up to 3 log_10_ units).

### 2.3. Acquired Tolerance to aBL Was Stable

**Figure 4:**
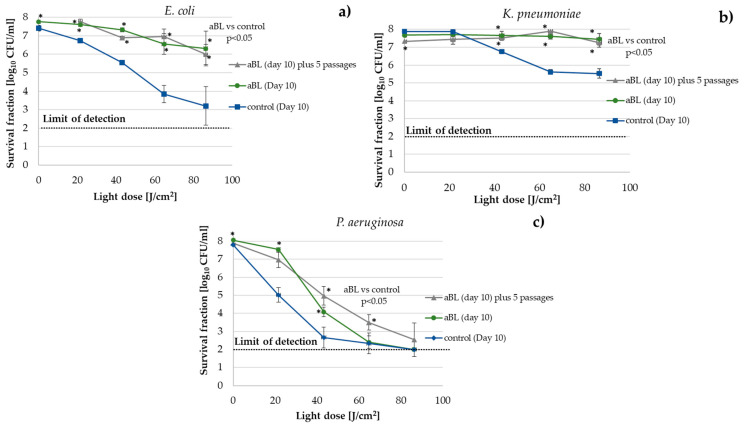
The phenotypic stability of the developed tolerance to aBL. The samples that originated from the 10th consecutive cycle of aBL treatment were transferred to 5 mL of fresh LB medium and cultured at 37 °C for 16–20 h. A total of 50-μL aliquots of the overnight cultures were transferred to tubes containing fresh LB medium. The cycle of transfer—regrowth—transfer was repeated five times. After five passage cycles, the cultures were diluted to an OD of 0.5 McF, and 100 μL of the bacterial suspensions were irradiated with 415 nm light at a dose up to 86.4 J/cm^2^ for *E. coli* (**a**), *K. pneumoniae* (**b**), and *P. aeruginosa* (**c**). The resulting suspensions were compared with the initial samples and with the untreated controls. The detection limit was 100 CFU/mL. The values are the means of three separate experiments. The error bars represent the standard deviation (SD). * All of the significant differences were estimated in comparison to the control (Day 10).

During the assessment of the risk of tolerance/resistance development, the phenotypic stability of the developed adaptation was assessed. *E. coli*, *K. pneumoniae*, and *P. aeruginosa* cultures that originated from the 10th consecutive cycle that were expressing significant tolerance to aBL were passaged for the subsequent five cycles without selection pressure. Subsequently, the susceptibility of the passaged *E. coli*, *K. pneumoniae*, and *P. aeruginosa* cultures to aBL was investigated and compared with the susceptibility of the respective cultures that originated directly from the 10th cycle of treatment. No loss of the developed aBL tolerance was observed for each tested strain (Figure 4). The obtained results confirmed the presumption that the developed tolerance results from genetic alterations were induced by multiple sublethal aBL irradiations.

### 2.4. E. coli tolC-, tolA-, umuD- and recA-Deficient Mutants Reveal Possible Mechanisms for aBL Tolerance Development

To confirm the importance of the *tolC*, *tolA*, *umuD*, and *recA* genes in the bacterial response to aBL, we subjected *tolC*-, *tolA*-, *umuD*-, and *recA*-deficient mutants (KEIO collection, NIG, Japan) to multiple sublethal exposures of aBL under conditions that were identical to those that were applied to the wild-type *E. coli* K-12 strain.

**Figure 5:**
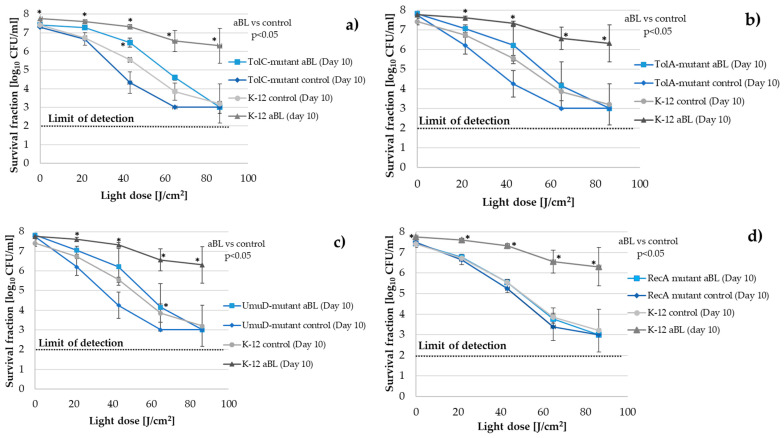
*E. coli* K-12 *tolC*-, *tolA*-, *umuD*-, and *recA-*deficient mutant responses to sublethal aBL treatment. The *tolC*-, *tolA*-, *umuD*-, and *recA*-deficient mutants were subjected to multiple sublethal exposures of aBL under conditions that were identical to those that were applied to the wild-type *E. coli* K-12 strain. (**a**) The *tolC*-deficient mutant developed weaker tolerance to aBL than the wild-type strain, similarly to the *tolA*- *(***b**) and *umuD*-deficient mutant (**c**); (**d**) The lack of a RecA protein prevented the development of tolerance to aBL. * All of the significant differences were estimated in comparison to the control from each day.

When the results of the ten treatment cycles were compiled, we observed that: (i) The *tolC*-, *tolA*-, and *umuD-*deficient mutants were significantly more sensitive to aBL than the wild-type strain (after 10 aBL inactivation cycles, the control passaged without selection pressure showed a greater decrease in CFU/mL survival compared to the wild-type control); (ii) The *tolC*-, *tolA*-, and *umuD-*deficient mutants developed significantly weaker tolerance than the wild-type *E. coli* K-12 strain. For example, at a dose of 64.8 J/cm^2^, the difference in response was more than a 2 log_10_ decrease in survival compared to the tolerant wild-type strain. aBL at the dose of 86.4 J/cm^2^ caused eradication of the *tolC*-, *tolA*-, and *umuD-*deficient mutants, comparable to the passaged control (Figure 5a–c, retrospectively); and (iii) The *recA*-deficient mutant did not develop tolerance under multiple sublethal aBL treatments (Figure 5d).

## 3. Discussion

Multidrug-resistant Gram-negative bacteria, mainly *Enterobacteriaceae*, *P. aeruginosa*, and *A. baumannii*, are significant challenges for global healthcare. Therefore, novel and alternative approaches are urgently needed to overcome this global health crisis. One such innovative non-antibiotic approach is aBL inactivation. Recently, aBL effectiveness and mechanisms of action have been extensively studied. The results that were obtained by Dai et al. (2013) showed that aBL treatment (415 nm) led to efficient inactivation (>7 log_10_ CFU/mL) of *P. aeruginosa* in vitro and in vivo [4]. McKenzie et al. (2013) reported a decrease (up to 5–7 log_10_) in the survival rate of *P.aeruginosa* biofilms after aBL treatment (405 nm) [6]. Vollmerhausen et al. (2017) showed a reduction in cell viability (approx. 3 log_10_ CFU/mL) after the treatment of a uropathogenic *E. coli* biofilm with 420 nm aBL [7]. Halstead et al. (2016) also demonstrated the high efficiency (≥5-log_10_ decrease in cell viability) of aBL (400 nm) against Gram-negative nosocomial pathogens, including *A. baumannii*, *Stenotrophomonas maltophilia*, *P. aeruginosa*, *E. coli*, and *K. pneumoniae*. The results showed that Gram-negative microbes are less susceptible to aBL phototreatment than Gram-positive microbes [8]. Additionally, equal efficacies against drug-resistant and drug-sensitive specimens were observed [9]. An in vivo study by Wang et al. (2016) demonstrated that exposure of mouse wounds, that were infected by *A. baumannii*, to aBL caused an imposed reduction in CFU (approx. 3 log_10_ CFU/mL) [10]. The results that are cited above show that aBL has substantial bactericidal efficacy against multidrug-resistant pathogens and could contribute to the urgent need for new antimicrobial therapies.

The efficacy of aBL treatment is due to its multi-target mode of action, but its mechanisms of action remain unclear. The main hypothesized mechanism involves the presence of photoactive endogenous compounds such as protoporphyrin, coproporphyrin, and uroporphyrin, which occur naturally in bacterial cells [16,17]. Endogenous porphyrins absorb Soret-band light (405–420 nm), which induces excitation to the triplet state and singlet oxygen generation [17]. During its very short half-life, the singlet oxygen attacks nearby molecules and produces other reactive oxygen species (ROS), such as super-oxides, peroxides, and hydroxyl radicals. ROS is toxic to cell structures and can cause lethal damage, such as oxidation of proteins and DNA, which has an impact on the denaturation and inactivation of essential enzymes [3,26]. The results that were obtained by Wu et al. (2018) suggest that the cell membrane is a major target of ROS during aBL irradiation, leading to alterations in the lipid profiles, particularly the unsaturated fatty acid components [27]. In addition to lipids, aBL affects membrane proteins and decreases adhesion, information reception, and transmembrane transport ability [28]. Moreover, blue light may cause the leakage of K^+^, Mg^2+^, and Ca^2+^ ions outside of the cell, altering the transmembrane potential [27,29].

During the development of new antimicrobial approaches, the risk of tolerance and/or resistance should be assessed. There is no standard protocol to predict the bacterial resistance development to photodynamic inactivation. Therefore, we proposed that the tolerance/resistance study protocol should be based on the following requirements: (I) the subculture should originate from the treated suspension instead of a single surviving colony; (II) the treatment condition should result in reduction to retain sufficient survivors (doses that are equal to the MDK_90-99_ or resulting in an approximately 1–2 log_10_ unit reduction in viable counts); (III) sequential subculturing and treatment should be conducted for up to 10–20 cycles; (IV) phenotypic stability testing should be performed; and (V) untreated controls should be simultaneously passaged under the same conditions.

**Figure 6:**
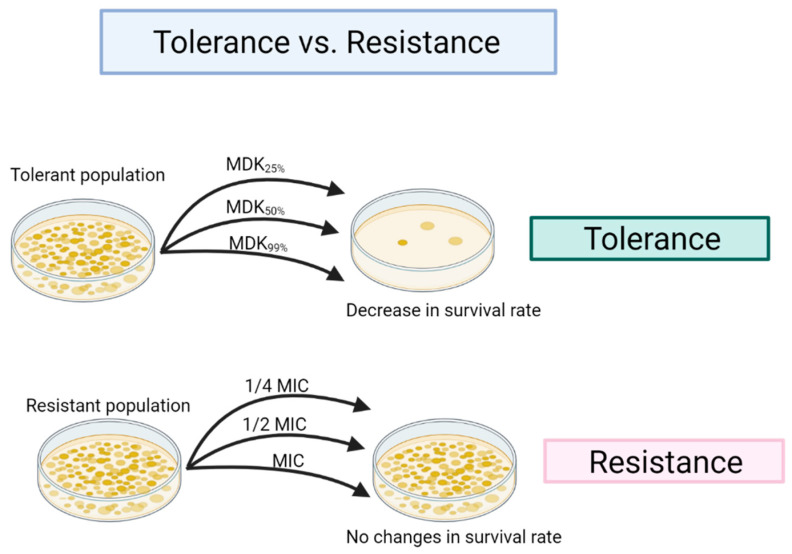
The differences between resistance and tolerance. Resistance refers to an inherited decrease in antimicrobial effectiveness and is usually determined using minimum inhibitory concentration (MIC) testing, while tolerance, which is also associated with treatment failure, cannot be measured with such testing because tolerant specimens could have the same MIC values as susceptible strains. An effective tool to quantify the tolerance is the minimal duration to kill 99% of cells (MDK_99_). A higher MDK_99_ indicates a longer treatment time to obtain the same level of killing as in susceptible strains [18,19]. The phenomenon of tolerance differs from resistance mainly in the employment of more rigorous experimental conditions (i.e., higher light doses result in bacterial eradication). In the case of resistance, the use of even higher treatment doses does not cause a killing effect.

Recently, some reports have indicated that the development of microbial tolerance to aBL could be initiated by sublethal phototreatment. At this point, the distinction between tolerance and resistance should be explained (Figure 6). Resistance, when referring to antibiotics, is defined as an acquired and inherited decrease in the effectiveness of a given antimicrobial that results in the need for higher concentrations of the drug, whereas tolerance can be overcome by, for example, a longer treatment duration to achieve the same killing efficiency. It is worth mentioning that the development of resistance to aBL has not yet been described, which is a very promising sign and proves the advantage of phototherapy over antibiotic therapy in the context of the development of potential resistance.

**Figure 7:**
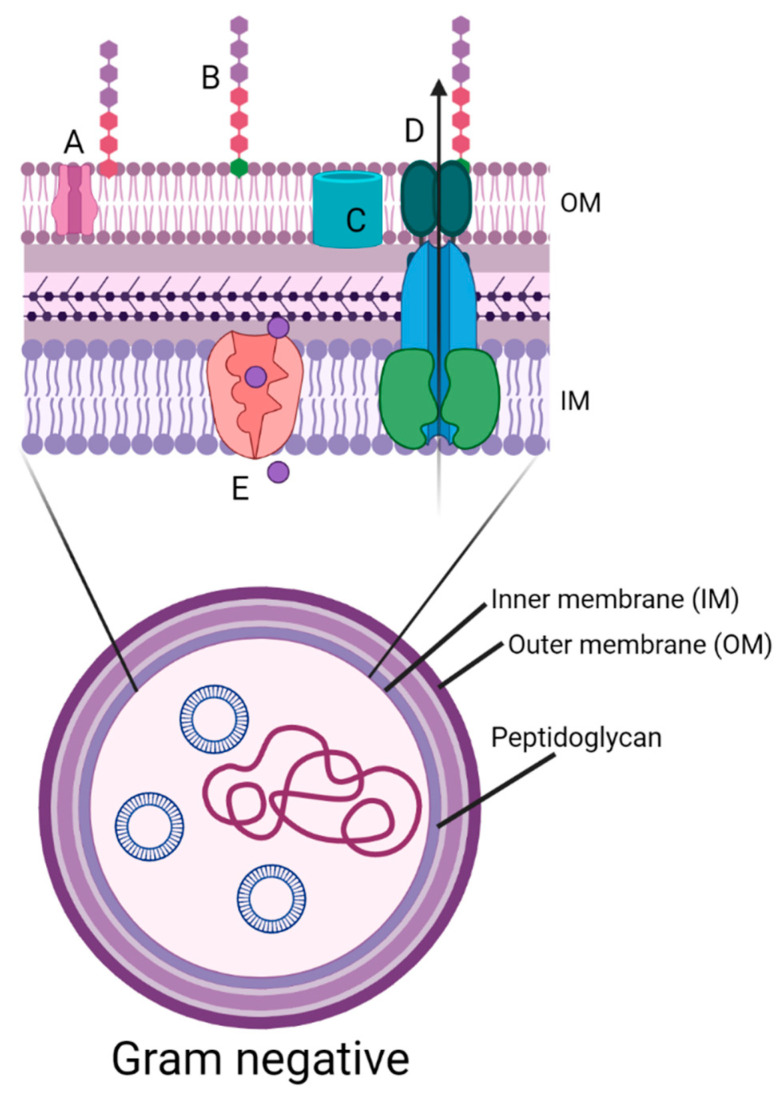
Diagram of the cell wall structure of a Gram-negative bacterium. Gram-negative bacteria possess an envelope that is composed of three layers. The first layer is the outer membrane (OM) The OM is composed of phospholipids, lipoproteins (A), lipopolysaccharide (LPS) (B) and proteins such as porins (C). The peptidoglycan cell wall is the second layer. The third layer is the cytoplasmic or inner membrane (IM). The IM is a phospholipid bilayer responsible for, inter alia, transport (D- efflux pumps) and sodium-potassium ions (E) [30]. The presence of the outer and inner membrane leads to decreased aPDI effectiveness against Gram-negative bacteria [31].

In this study, we confirmed that the application of the protocol that we proposed in our previous study allowed us to observe the development of tolerance in not only Gram-positive (*S. aureus*, *E. faecium*, and *S. agalactiae*) [20] but also Gram-negative bacteria (*E. coli*, *K. pneumoniae*, and *P. aeruginosa*). The observed adaptation is not surprising, as Gram-negative bacteria are less sensitive to photodynamic inactivation due to the composition and morphology of the cell wall. Gram-negative bacteria possess an envelope that is composed of three layers (Figure 7). The first protective layer, which is not present in Gram-positive bacteria, is the outer membrane (OM). The OM is composed of phospholipids, lipopolysaccharide (LPS), proteins such as porins, and other molecules. The peptidoglycan cell wall is the second layer. The third layer is inner membrane (IM). The IM is a phospholipid bilayer responsible for, inter alia, cell transport [30].

Gram-negative bacteria can rapidly develop adaptive responses to many environmental stressors. Transcriptional and translational regulations are crucial for survival in dynamic environments. Moreover, microbes have evolved numerous mechanisms at the membrane structure level to cope with physiochemical stress and also adapt to it [32]. One tolerance mechanism are multidrug efflux pumps that act against various antibiotics, toxins, or solvents. For instance, Aono and Kobayashi (1997) observed alterations in the membrane components and a low cell hydrophobicity in organic solvent-tolerant mutants that were derived from *E. coli* K-12 [33]. The major organic solvent tolerance mechanism is the effective removal from the cytoplasm with the aid of efflux pumps [34]. Overexpression of the efflux pumps can also result in a decreased susceptibility to antimicrobials. For instance, *P. aeruginosa* exhibits innate resistance to several antibiotics. Another mechanism that can lead to adaptation are permeability defects that change the envelope permeability. Proteins that are involved in the transport of molecules across the outer membrane are called porins. Mutations in genes that encode porins may initiate resistance development. Furthermore, the loss of LPS may drive decreased membrane integrity and can cause adaptation [25].

The potential development of bacterial resistance to various DNA damaging factors (e.g., radiation) has been concerning scientists for many decades. Witkin (1946) observed a stable, heritable increase in resistance to ultraviolet radiation (UV) and X-rays in *E. coli* [35] and suggested that radiation resistance occurs as a result of spontaneous mutation [36]. Harris el al. (2009) noticed high radiation resistance in radio-sensitive *E. coli* K-12 after treatment with high doses of ionizing radiation. Scientists performed 20 subsequent cycles of ^60^Co irradiation and re-growth with increasing doses of exposure during the cycles progress (each exposure was adjusted to kill >99% of the cells). The adaptation was stable. Moreover, radio-resistant strains were 10 times more resistant to UV irradiation when compared to the wild-type strain. The mutation in the *recA* gene was commonly observed for these strains, also additional *recA* alleles were detected. The authors suggested that the *recA* mutations have some contribution to radiation adaptation [37]. Lage et al. (2000) studied the interactions between visible or infrared (IR) radiation and UV (254 nm) radiation in *E. coli* K12 strain. Pre-irradiation with 15 or 30 min doses of monochromatic visible light (indigo (446 nm), blue (466 nm), green (570), and red (685 nm)) and with polychromatic red and IR radiation led to decreased cell sensitivity after subsequent irradiation with UV light. Moreover, IR pre-treatment increased the tolerance to subsequent lethal heat (51 °C), probably due to an IR-induced heat-shock response. The authors suggested that low doses of visible light caused some DNA damage and triggered DNA repair mechanisms, which contributed to the fact that the cells were more resistant to other factors (i.e., UV) [38]. Guffey et al. (2013) observed a decrease in treatment effectiveness in *S. aureus* after five cycles of irradiation with a low dose (9 J/cm^2^) of aBL (405 nm). The adaptation that was observed by the authors was suggested to be that bacteria may be capable of developing tolerance to blue light irradiation [39], which corresponds to the results of our previous and current research.

The mechanism of the development of tolerance to aBL in Gram-negative bacteria remains unknown. A probable mechanism is the participation of efflux pumps and membrane channel-tunnel proteins, such as TolC. TolC is a member of the outer membrane efflux protein (OEP) family that is involved in preventing toxins, dyes, antibiotics, detergents, and other harmful molecules from crossing the OM of Gram-negative bacteria [40]. TolC plays a significant role in the adaptation of bacteria to unfavorable environments, thus, inactivation of the *tolC* gene affects the virulence and physiology of *Enterobacteriaceae*, making cells more susceptible to antibacterial agents [41]. The *P. aeruginosa* genome also contains OEPs. AprF and OpmH are very closely related to *E. coli* TolC [42]. *E. coli* TolC is a member of several regulons that promote resistance to numerous antibiotics and super-oxides (i.e., marA/soxS/rob regulon) and is constitutively expressed; however, its expression can be upregulated in response to different environmental signals [43,44]. TolC is the principal factor in protection against redox-active antibiotics that kill bacteria by reactive oxygen species (ROS) generation, which can lead to membrane damage [45]. Turlin et al. (2014) investigated the mechanism of protoporphyrin IX (PPIX) efflux and observed that the MaxcAB-TolC pump was a major pump involved in PPIX efflux. They confirmed that PPIX accumulation in *macAB* and *tolC* mutants led to increased photosensitivity [46]. The overproduction of intracellular porphyrins in the presence of oxygen and light is potentially toxic due to ROS production. The endogenous coproporphyrin (a precursor of PPIX) produces the majority of free radicals [16,47]. Tatsumi and Wachi (2008) found that *tolC E. coli* mutants showed an improved sensitivity to exogenous 5-aminolevulinic acid (ALA) and accumulated large amounts of porphyrinogens and porphyrins intracellularly, while wild-type cells produced them extracellularly. Protoporphyrin (ogen) acted as a photosensitizer (PS). The *tolC* mutant accumulated mainly coproporphyrin (ogen), which also functioned as a PS [48]. The *tolC*-deficient mutant was significantly more sensitive to aBL than the wild-type strain and showed a greater decrease in CFU/mL survival than the wild-type control after 10 aBL treatment cycles. Moreover, the *tolC*-deficient mutant developed significantly weaker tolerance than the wild-type *E. coli* K-12 strain (an approximately 2 log_10_ decrease in survival compared to the tolerant wild-type strain). A dose of 86.4 J/cm^2^ caused the eradication of the *tolC-*deficient mutant, comparable to the passaged control (without the selective pressure of aBL) (Figure 5a). TolA is a cytoplasmic membrane protein that interacts with the *E. coli* porins (e.g., OmpF) and it is crucial for the functionality and stability of the *E. coli* outer membrane [49]. The *E. coli* *tolA* gene contains a highly variable tandem repeats (TR) region [50,51]. The results that were obtained by Zhou et al. (2012) suggest that the size of the *tolA* TR region could contribute to the fitness of *E. coli* under specific stress conditions, thereby influencing its tolerance [51]. Similarly to the *tolC-*deficient mutant, the *tolA*-deficient mutant was significantly more sensitive to aBL than the wild-type strain and showed a greater decline in CFU/mL survival than the wild-type control after 10 passages. Furthermore, the *tolA*-deficient mutant did not develop a significant tolerance compared to the wild-type *E. coli* K-12 strain (Figure 5b).

Stress-induced mutagenesis (SIM) may drive the process of bacterial adaptation during stress responses. SIM plays a role in increased genetic variability and leads to potentially beneficial mutations when bacteria are subjected to stressors, such as oxidation and irradiation. Adaptationists postulate that bacteria enhance their potential for adaptation by modulating the rate of mutation, so mutations are, essentially, the price bacteria have to pay for survival [52]. Blue light has mutagenic potential and could trigger a repair response. McGinty and Fowler (1985) showed base-pair substitution (transversions at both G:C and A:T sites) and frameshift mutations in *E. coli* that were induced by visible light (450 nm) [53]. *E. coli* PolV SOS (umuD′ 2 C complex) polymerase is the main factor that leads to mutagenesis [54]. Inactivation of the SOS response by the inhibition of *recA* is considered a possible therapeutic adjuvant in combined therapy to reduce the ability of bacteria to produce antibiotic-resistant mutants [55]. In a previous study, we demonstrated that sublethal aBL and aPDI led to substantial DNA damage in *S. aureus* by the activation of *recA* and an increased SOS response [56]. Moreover, in our previous report on tolerance development in Gram-positive species (*S. aureus*, *S. agalactiae*, and *E. faecium*), we observed an increased mutation rate (rifampicin-resistant (RIF^R^) mutant selection test), increased stress-responsive error-prone DNA polymerase V gene expression, and a lack of tolerance development in *recA*- and *umuC-*deficient mutants of *S. aureus*, which suggested a possible mechanism for adaptation development. In this study, we also hypothesized that tolerance development should be expected in Gram-negative bacteria. In the current study, the *recA*- and *umuD-*deficient mutant of *E. coli* K-12 was subjected to multiple sublethal aBL treatments, and the obtained results indicated that the lack of the RecA and UmuD proteins prevented the development of tolerance to aBL; both the *recA*- and *umuD-*deficient mutants showed no development of adaptation to photoinactivation (Figure 5c,d). This corresponds with our previously obtained results regarding *S. aureus* tolerance development [20]. A very recent study that was conducted by Snell et al. (2021) resulted in selective adaption of *S. aureus* to methylene blue-mediated aPDI (MB-aPDI). The authors performed global genome and transcriptome analyses to identify the regulatory and genetic adaptations that contributed to the observed tolerance development. In a global cellular response to aPDI, the scientists identified, among other things, DNA replication, recombination, and repair; oxidative response; and membrane and cell wall biogenesis. The genome analysis of aPDI-tolerant *S. aureus* strain revealed a nonsynonymous mutation in the transcriptional repressor, which mediates the oxidant response (QsrR). Moreover, an obtained tolerance to MB-aPDI was associated with superoxide dismutase and the global methylhydroquinone (MHQ)-quinone transcriptome network [22].

aBL and aPDI are still considered as low-risk treatments for tolerance and resistance development. The discrepancies in the results that were obtained by various research groups raise the question of the need for standardized protocols for the testing of bacterial resistance to light-based therapies, which was also highlighted in the latest review by Marasini et al. (2021). The authors suggested that in future research, it is important to assess reproducibility during assessment of the potential development of tolerance or resistance, and we agree with this statement [57]. The literature shows that Gram-negative bacteria, including *E. coli*, have repeatedly acquired adaptations to many chemical factors, such as acids [58,59], organic solvents [33,34], heavy metals [60,61], and physical factors such as ionizing [37] or UV radiation [35]. Therefore, it cannot be completely excluded that bacteria could develop an adaptation to visible light (i.e., aBL) or photodynamic inactivation (aPDI) due to DNA damage and the triggering of repair mechanisms.

## 4. Materials and Methods

### 4.1. Strains and Culture Conditions

Analyses were performed using the following reference strains: *E. coli* K-12 (Dharmacon™, Lafayette, CO, USA), *K. pneumoniae* ATCC700603, and *P. aeruginosa* PA14. All of the strains were cultured in LB medium (BTL, Łódź, Poland) at 37 °C under aerobic conditions. The samples from cycles 1, 5, 10, and 15 were stored at −80 °C, and before use in experiments, they were freshly inoculated into new LB medium and incubated overnight (16–20 h) in an orbital incubator (Innova 40, Brunswick, Germany) at 150 rpm. Assays involving *E. coli* K-12 single-gene mutants (KEIO collection, NIG, Mishima, Shizuoka, Japan) were carried out with the addition of kanamycin (15 µg/mL) [62].

### 4.2. Light Source

Irradiation was performed with a light-emitting diode (LED) light source that emitted blue light (λmax 415 nm, irradiance of 25 mW/cm^2^) (Cezos, Gdynia, Poland).

### 4.3. Determination of the aBL Minimal Duration to Kill 99% of Cells (MDK_99_)

All of the strains were inoculated in triplicate and cultured at 37 °C in LB for 16–20 h. Then, the cultures were adjusted to an optical density (OD) of 0.5 McF (approx. 5 × 10^7^ CFU/mL), and the aliquots were transferred to 96-well microtiter plates (100 µL per well). The aBL samples were illuminated without shaking with different light doses of 415 nm blue light (21.6 J/cm^2^, 43.2 J/cm^2^, 64.8 J/cm^2^, and 86.4 J/cm^2^, which corresponded to an exposure time of 15, 30, 45, and 60 min, respectively)). After irradiation, 10 µL aliquots were serially diluted tenfold in phosphate-buffered saline (PBS) to generate dilutions of 10^−1^ to 10^−4^ and streaked horizontally on LB agar plates (BTL, Łódź, Poland). The LB agar plates were incubated at 37 °C for 16–20 h, and then the colonies were counted to estimate the survival rate. The control groups included cells that were not treated with blue light.

### 4.4. Determination of Tolerance Development Following Repeated Sublethal Exposure to aBL

**Figure 8:**
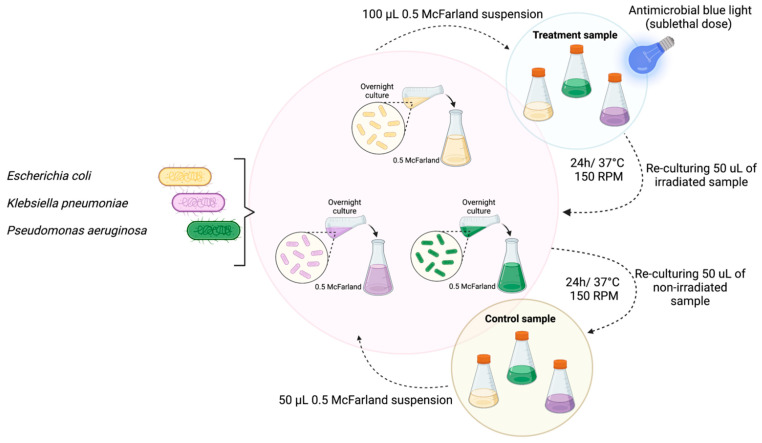
Experimental workflow. All Gram-negative representative bacteria (*E. coli*, *K. pneumoniae*, and *P. aeruginosa*) were inoculated and cultured at 37 °C in LB medium for 16–20 h. The cultures were diluted to an OD of 0.5 McF, and 100-µL bacterial suspension samples were irradiated with 415 nm light at light doses close to the MDK_99_. Following exposure, 50 μL of the treated suspension was transferred into fresh LB medium (5 mL) to regrow overnight. The next day, after 16–20 h of incubation, the treatment was repeated under the same conditions. The cycle of exposure—regrowth—exposure was repeated 15 times. Control groups represent the cells that were not treated with blue light but passaged daily. Subsequently, all of the samples that originated from the 1st, 5th, 10th, and 15th cycles were tested for potential tolerance development.

All of the strains were inoculated in triplicate and cultured at 37 °C in LB for 16–20 h. Then, the cultures were diluted to an OD of 0.5 McF. Bacterial suspension sample aliquots of 100 µL were irradiated with 415 nm light at light doses close to the MDK_99_. Following exposure, 10-μL aliquots of the treated samples were collected to determine the survival rate. Sample aliquots of 50 μL were transferred into fresh LB medium (5 mL) to regrow overnight. The next day, after 16–20 h of incubation, the treatment was repeated under the same conditions. The cycle of exposure—regrowth—exposure was repeated 15 times. Potential reductions in the susceptibility to aBL were examined after the 5th, 10th, and 15th consecutive cycles at higher doses of light (up to 86.4 J/cm^2^). The experimental workflow is presented in Figure 8.

### 4.5. Stability of the Acquired Tolerance to aBL

The experiments were performed using the samples that were taken from the consecutive aBL treatment cycle in which a significant decrease in susceptibility was observed (10th consecutive cycle) and transferred to fresh LB medium and cultured overnight. The cycles of transfer—regrowth—transfer were repeated 5 times. On the appropriate cycle, the cultures were diluted to an OD of 0.5 McF, and 100 μL of the bacterial suspensions were irradiated with 415 nm light at a dose up to 86.4 J/cm^2^. The resulting suspensions were compared with the initial samples and with the untreated controls.

### 4.6. Statistical Analysis

The statistical analyses were performed using Excel. The quantitative variables were characterized by the arithmetic mean of the standard deviation. Statistical significance of the differences between the two groups were processed with the Student’s *t*-test. *p* values that were less than 0.05 were considered statistically significant.

## 5. Conclusions

In the current study, we proved that tolerance development is not unlikely but possible, which is contrary to the consensus that is reached by most authors who are concerned about the issue. Our team observed the development of tolerance to aBL in each species that was subsequently irradiated (*E. coli*, *K. pneumoniae*, and *P. aeruginosa*). The observed adaptations were stable features in each case, which could mean that they were most likely the result of genetic alterations that were induced by multiple sublethal treatments. A potential mechanism explaining the observed adaptation may be mutations that are driven by SOS mutagenesis that is related to the genes of the efflux pump proteins (such as the aforementioned TolC). Mutations leading to the overexpression of genes that are responsible for the active pumping of endogenous photosensitizing compounds out of the cell could allow bacteria to survive longer exposure times to aBL. The mechanism of tolerance development remains unclear, however, the results of studies involving *E. coli* KEIO mutants [52] confirm that the mechanism leading to the adaptation is complex and dependent on more than one factor.

It should be mentioned that resistance to photoinactivation (both aBL and aPDI) has not been observed thus far, and the observed tolerance development does not disqualify aBL as a potential treatment for infection with multidrug-resistant strains. The lack of resistance development in this context demonstrates the superiority of phototherapy over antibiotics. The observed tolerance indicates only the potential limitations that should be overcome when sustainable solutions are applied.

## Figures and Tables

**Table 1:** aBL treatment conditions.

Reference Strain	Light Conditions [J/cm^2^]	Decrease in Viable Count (log_10_ CFU/mL)
*E. coli* K-12	32.4 (100% LED power)	1.2
*K. pneumoniae* ATCC 700603	61.2 (75% LED power)	2.2
*P. aeruginosa* P14	6.4 (75% LED power)	1.2

## Data Availability

The data presented in this study are available on request from the corresponding author.

## References

[1] O’Nell J. (2016). Tackling Drug-Resistant Infections Globally: Final Report and Recommendations. The Review on Antimicrobial Resistance.

[2] Tacconelli E., Carrara E., Savoldi A., Harbarth S., Mendelson M., Monnet D.L., Pulcini C., Kahlmeter G., Kluytmans J., Carmeli Y. (2018). Discovery, research, and development of new antibiotics: The WHO priority list of antibiotic-resistant bacteria and tuberculosis. Lancet Infect. Dis..

[3] Dai T. (2017). The antimicrobial effect of blue light: What are behind?. Virulence.

[4] Dai T., Gupta A., Huang Y., Yin R., Murray C.K., Vrahas M.S., Sherwood M.E., Tegos G.P., Hamblin M.R. (2013). Blue light rescues mice from potentially fatal *Pseudomonas aeruginosa* burn infection: Efficacy, safety, and mechanism of action. Antimicrob. Agents Chemother..

[5] Dai T., Gupta A., Huang Y.Y., Sherwood M.E., Murray C.K., Vrahas M.S., Kielian T., Hamblin M.R. (2013). Blue light eliminates community-acquired methicillin-resistant *Staphylococcus aureus* in infected mouse skin abrasions. Photomed. Laser Surg..

[6] McKenzie K., Maclean M., Timoshkin I.V., Endarko E., MacGregor S.J., Anderson J.G. (2013). Photoinactivation of bacteria attached to glass and acrylic surfaces by 405 nm light: Potential application for biofilm decontamination. Photochem. Photobiol..

[7] Vollmerhausen T.L., Conneely A., Bennett C., Wagner V.E., Victor J.C., O’Byrne C.P. (2017). Visible and UVA light as a potential means of preventing *Escherichia coli* biofilm formation in urine and on materials used in urethral catheters. J. Photochem. Photobiol. B Biol..

[8] Halstead F.D., Thwaite J.E., Burt R., Laws T.R., Raguse M., Moeller R., Webber M.A., Oppenheim B.A. (2016). Antibacterial activity of blue light against nosocomial wound pathogens growing planktonically and as mature biofilms. Appl. Environ. Microbiol..

[9] Enwemeka C.S., Williams D., Hollosi S., Yens D., Enwemeka S.K. (2008). Visible 405 nm SLD light photo-destroys methicillin-resistant *Staphylococcus aureus* (MRSA) in vitro. Lasers Surg. Med..

[10] Wang Y., Wu X., Chen J., Amin R., Lu M., Bhayana B., Zhao J., Murray C.K., Hamblin M.R., Hooper D.C. (2016). Antimicrobial blue light inactivation of gram-negative pathogens in biofilms: In vitro and in vivo studies. J. Infect. Dis..

[11] Nakonieczna J., Wozniak A., Pieranski M., Rapacka-Zdonczyk A., Ogonowska P., Grinholc M. (2019). Photoinactivation of ESKAPE pathogens: Overview of novel therapeutic strategy. Future Med. Chem..

[12] Hoenes K., Bauer R., Meurle T., Spellerberg B., Hessling M. (2021). Inactivation Effect of Violet and Blue Light on ESKAPE Pathogens and Closely Related Non-pathogenic Bacterial Species—A Promising Tool Against Antibiotic-Sensitive and Antibiotic-Resistant Microorganisms. Front. Microbiol..

[13] Glaeser J., Nuss A.M., Berghoff B.A., Klug G. (2011). Singlet oxygen stress in microorganisms. Adv. Microb. Physiol..

[14] Maisch T. (2015). Resistance in antimicrobial photodynamic inactivation of bacteria. Photochem. Photobiol. Sci..

[15] Wang Y., Wang Y., Wang Y., Murray C.K., Hamblin M.R., Hooper D.C., Dai T. (2017). Antimicrobial blue light inactivation of pathogenic microbes: State of the art. Drug Resist. Updates.

[16] Amin R.M., Bhayana B., Hamblin M.R., Dai T. (2016). Antimicrobial blue light inactivation of *Pseudomonas aeruginosa* by photo-excitation of endogenous porphyrins: In vitro and in vivo studies. Lasers Surg. Med..

[17] Dai T., Hamblin M.R. (2017). Visible blue light is capable of inactivating *Candida albicans* and other fungal species. Photomed. Laser Surg..

[18] Fridman O., Goldberg A., Ronin I., Shoresh N., Balaban N.Q. (2014). Optimization of lag time underlies antibiotic tolerance in evolved bacterial populations. Nature.

[19] Brauner A., Fridman O., Gefen O., Balaban N.Q. (2016). Distinguishing between resistance, tolerance and persistence to antibiotic treatment. Nat. Rev. Microbiol..

[20] Rapacka-Zdonczyk A., Wozniak A., Pieranski M., Woziwodzka A., Bielawski K.P., Grinholc M. (2019). Development of *Staphylococcus aureus* tolerance to antimicrobial photodynamic inactivation and antimicrobial blue light upon sub-lethal treatment. Sci. Rep..

[21] Pieranski M., Sitkiewicz I., Grinholc M. (2020). Increased photoinactivation stress tolerance of *Streptococcus agalactiae* upon consecutive sublethal phototreatments. Free Radic. Biol. Med..

[22] Snell S.B., Gill A.L., Haidaris C.G., Foster T.H., Baran T.M., Gill S.R. (2021). *Staphylococcus aureus* Tolerance and Genomic Response to Photodynamic Inactivation. Msphere.

[23] Leanse L.G., Harrington O.D., Fang Y., Ahmed I., Goh X.S., Dai T. (2018). Evaluating the Potential for Resistance Development to Antimicrobial Blue Light (at 405 nm) in Gram-Negative Bacteria: In vitro and in vivo Studies. Front. Microbiol..

[24] Zhang Y., Zhu Y., Gupta A., Huang Y., Murray C.K., Vrahas M.S., Sherwood M.E., Baer D.G., Hamblin M.R., Dai T. (2014). Antimicrobial blue light therapy for multidrug-resistant *Acinetobacter baumannii* infection in a mouse burn model: Implications for prophylaxis and treatment of combat-related wound infections. J. Infect. Dis..

[25] Breijyeh Z., Jubeh B., Karaman R. (2020). Resistance of Gram-negative bacteria to current antibacterial agents and approaches to resolve it. Molecules.

[26] Cieplik F., Späth A., Leibl C., Gollmer A., Regensburger J., Tabenski L., Hiller K.-A., Maisch T., Schmalz G. (2014). Blue light kills *Aggregatibacter actinomycetemcomitans* due to its endogenous photosensitizers. Clin. Oral Investig..

[27] Wu J., Chu Z., Ruan Z., Wang X., Dai T., Hu X. (2018). Changes of intracellular porphyrin, reactive oxygen species, and fatty acids profiles during inactivation of methicillin-resistant *Staphylococcus aureus* by antimicrobial blue light. Front. Physiol..

[28] Alves E., Faustino M.A., Neves M.G., Cunha A., Tome J., Almeida A. (2014). An insight on bacterial cellular targets of photodynamic inactivation. Future Med. Chem..

[29] Boucher H.W., Talbot G.H., Bradley J.S., Edwards J.E., Gilbert D., Rice L.B., Scheld M., Spellberg B., Bartlett J. (2009). Bad bugs, no drugs: No ESKAPE! An update from the Infectious Diseases Society of America. Clin. Infect. Dis..

[30] Silhavy T.J., Kahne D., Walker S. (2010). The bacterial cell envelope. Cold Spring Harb. Perspect. Biol..

[31] Rapacka-Zdończyk A., Woźniak A., Michalska K., Pierański M., Ogonowska P., Grinholc M., Nakonieczna J. (2021). Factors determining the susceptibility of bacteria to antibacterial photodynamic inactivation. Front. Med..

[32] Ramos J.L., Duque E., Gallegos M.-T., Godoy P., Ramos-González M.I., Rojas A., Terán W., Segura A. (2002). Mechanisms of solvent tolerance in gram-negative bacteria. Annu. Rev. Microbiol..

[33] Aono R., Kobayashi H. (1997). Cell surface properties of organic solvent-tolerant mutants of *Escherichia coli* K-12. Appl. Environ. Microbiol..

[34] Li X.Z., Zhang L., Poole K. (1998). Role of the multidrug efflux systems of *Pseudomonas aeruginosa* in organic solvent tolerance. J. Bacteriol..

[35] Witkin E.M. (1947). Genetics of resistance to radiation in *Escherichia coli*. Genetics.

[36] Witkin E.M. (1946). Inherited differences in sensitivity to radiation in *Escherichia coli*. Proc. Natl. Acad. Sci. USA.

[37] Harris D.R., Pollock S.V., Wood E.A., Goiffon R.J., Klingele A.J., Cabot E.L., Schackwitz W., Martin J., Eggington J., Durfee T.J. (2009). Directed evolution of ionizing radiation resistance in *Escherichia coli*. J. Bacteriol..

[38] Lage C., Teixeira P.C.N., Leitao A.C. (2000). Non-coherent visible and infrared radiation increase survival to UV (254 nm) in *Escherichia coli* K12. J. Photochem. Photobiol. B Biol..

[39] Guffey J.S., Payne W., Jones T., Martin K. (2013). Evidence of resistance development by *Staphylococcus aureus* to an in vitro, multiple stage application of 405 nm light from a supraluminous diode array. Photomed. Laser Surg..

[40] Zgurskaya H.I., Krishnamoorthy G., Ntreh A., Lu S. (2011). Mechanism and function of the outer membrane channel TolC in multidrug resistance and physiology of enterobacteria. Front. Microbiol..

[41] Sulavik M.C., Houseweart C., Cramer C., Jiwani N., Murgolo N., Greene J., DiDomenico B., Shaw K.J., Miller G.H., Hare R. (2001). Antibiotic susceptibility profiles of *Escherichia coli* strains lacking multidrug efflux pump genes. Antimicrob. Agents Chemother..

[42] Jo J.T., Brinkman F.S., Hancock R.E. (2003). Aminoglycoside efflux in *Pseudomonas aeruginosa*: Involvement of novel outer membrane proteins. Antimicrob. Agents Chemother..

[43] Duval V., Lister I.M. (2013). MarA, SoxS and Rob of *Escherichia coli*–Global regulators of multidrug resistance, virulence and stress response. Int. J. Biotechnol. Wellness Ind..

[44] Tikhonova E.B., Zgurskaya H.I. (2004). AcrA, AcrB, and TolC of *Escherichia coli* form a stable intermembrane multidrug efflux complex. J. Biol. Chem..

[45] Kohanski M.A., Dwyer D.J., Hayete B., Lawrence C.A., Collins J.J. (2007). A common mechanism of cellular death induced by bactericidal antibiotics. Cell.

[46] Turlin E., Heuck G., Simões Brandão M.I., Szili N., Mellin J.R., Lange N., Wandersman C. (2014). Protoporphyrin (PPIX) efflux by the MacAB-TolC pump in *Escherichia coli*. Microbiologyopen.

[47] Nitzan Y., Salmon-Divon M., Shporen E., Malik Z. (2004). ALA induced photodynamic effects on gram positive and negative bacteria. Photochem. Photobiol. Sci..

[48] Tatsumi R., Wachi M. (2008). TolC-dependent exclusion of porphyrins in *Escherichia coli*. J. Bacteriol..

[49] Derouiche R., Gavioli M., Bénédetti H., Prilipov A., Lazdunski C., Lloubes R. (1996). TolA central domain interacts with *Escherichia coli* porins. EMBO J..

[50] Zhou K., Vanoirbeek K., Aertsen A., Michiels C.W. (2012). Variability of the tandem repeat region of the *Escherichia coli tolA* gene. Res. Microbiol..

[51] Zhou K., Michiels C.W., Aertsen A. (2012). Variation of intragenic tandem repeat tract of *tolA* modulates *Escherichia coli* stress tolerance. PLoS ONE.

[52] Tenaillon O., Denamur E., Matic I. (2004). Evolutionary significance of stress-induced mutagenesis in bacteria. Trends Microbiol..

[53] McGinty L.D., Fowler R.G. (1982). Visible light mutagenesis in *Escherichia coli*. Mutat. Res. Mol. Mech. Mutagen..

[54] Pham P., Rangarajan S., Woodgate R., Goodman M.F. (2001). Roles of DNA polymerases V and II in SOS-induced error-prone and error-free repair in *Escherichia coli*. Proc. Natl. Acad. Sci. USA.

[55] Pomerantz R.T., Goodman M.F., O’Donnell M.E. (2013). DNA polymerases are error-prone at RecA-mediated recombination intermediates. Cell Cycle.

[56] Grinholc M., Rodziewicz A., Forys K., Rapacka-Zdonczyk A., Kawiak A., Domachowska A., Golunski G., Wolz C., Mesak L., Becker K. (2015). Fine-tuning recA expression in Staphylococcus aureus for antimicrobial photoinactivation: Importance of photo-induced DNA damage in the photoinactivation mechanism. Appl. Microbiol. Biotechnol..

[57] Marasini S., Leanse L.G., Dai T. (2021). Can microorganisms develop resistance against light based anti-infective agents?. Adv. Drug Deliv. Rev..

[58] Benjamin M.M., Datta A.R. (1995). Acid tolerance of enterohemorrhagic *Escherichia coli*. Appl. Environ. Microbiol..

[59] Arnold C.N., McElhanon J., Lee A., Leonhart R., Siegele D.A. (2001). Global analysis of *Escherichia coli* gene expression during the acetate-induced acid tolerance response. J. Bacteriol..

[60] Nakajima H., Kobayashi K., Kobayashi M., Asako H., Aono R. (1995). Overexpression of the robA gene increases organic solvent tolerance and multiple antibiotic and heavy metal ion resistance in *Escherichia coli*. Appl. Environ. Microbiol..

[61] Kawane R.S. (2012). Studies on antibiotics and heavy metal resistance profiling of *Escherichia coli* from drinking water and clinical specimens. Biosci. Discov..

[62] Baba T., Ara T., Hasegawa M., Takai Y., Okumura Y., Baba M., Datsenko K.A., Tomita M., Wanner B.L., Mori H. (2006). Construction of *Escherichia coli* K-12 in-frame, single-gene knockout mutants: The Keio collection. Mol. Syst. Biol..

